# Probabilistic inference of epigenetic age acceleration from cellular dynamics

**DOI:** 10.1038/s43587-024-00700-5

**Published:** 2024-09-23

**Authors:** Jan K. Dabrowski, Emma J. Yang, Samuel J. C. Crofts, Robert F. Hillary, Daniel J. Simpson, Daniel L. McCartney, Riccardo E. Marioni, Kristina Kirschner, Eric Latorre-Crespo, Tamir Chandra

**Affiliations:** 1 School of Informatics, University of Edinburgh, Edinburgh, UK; 2 MRC Human Genetics Unit, University of Edinburgh, Edinburgh, UK; 3 Institute of Ecology and Evolution, School of Biological Sciences, University of Edinburgh, Edinburgh, UK; 4 Centre for Genomic and Experimental Medicine, Institute of Genetics and Cancer, University of Edinburgh, Edinburgh, UK; 5 Cancer Research UK Scotland Institute, Glasgow, UK; 6 School of Cancer Sciences, University of Glasgow, Glasgow, UK; 7 Robert and Arlene Kogod Center on Aging, Mayo Clinic, Rochester, MN USA; 8 Department of Biochemistry and Molecular Biology, Mayo Clinic, Rochester, MN USA; 9 Department of Hematology, Mayo Clinic, Rochester, MN USA; 10 Department of Quantitative Health Sciences, Mayo Clinic, Rochester, MN USA

**Keywords:** Statistical methods, Medical research, Predictive markers, Stochastic modelling, Ageing

## Abstract

The emergence of epigenetic predictors was a pivotal moment in geroscience, propelling the measurement and concept of biological aging into a quantitative era; however, while current epigenetic clocks show strong predictive power, they are data-driven in nature and are not based on the underlying biological mechanisms driving methylation dynamics. We show that predictions of these clocks are susceptible to several confounding non-age-related phenomena that make interpretation of these estimates and associations difficult. To address these limitations, we developed a probabilistic model describing methylation transitions at the cellular level. Our approach reveals two measurable components, acceleration and bias, which directly reflect perturbations of the underlying cellular dynamics. Acceleration is the proportional increase in the speed of methylation transitions across CpG sites, whereas bias corresponds to global changes in methylation levels. Using data from 15,900 participants from the Generation Scotland study, we develop a robust inference framework and show that these are two distinct processes confounding current epigenetic predictors. Our results show improved associations of acceleration and bias with physiological traits known to impact healthy aging, such as smoking and alcohol consumption, respectively. Furthermore, a genome-wide association study of epigenetic age acceleration identified seven genomic loci.

## Abstract (web summary)

Epigenetic clocks estimate biological age; however, the underlying processes are incompletely understood. Here the authors developed a model that describes methylation changes at the cellular level to provide a biologically interpretable predictor of epigenetic age. They reveal acceleration and bias components, and links with health traits.

## Main

The role of age as the predominant risk factor for cancer, neurodegenerative disease and cardiovascular disease has motivated research into its underlying cellular mechanisms. Until recently, a major challenge in this field was the lack of a reliable method for accurately measuring biological age. A tipping point was the development of the first comprehensive epigenetic age predictors^1–4^. These models quantified biological age based on the presence of age-related changes in the DNA methylome of individuals. This development naturally led to the concept of epigenetic age acceleration, which is commonly defined, for an individual within a cohort, as the residual from the epigenetic clock’s predicted age and their chronological age^3,5^. The use of epigenetic clocks to measure the rate of epigenetic aging is now widely employed as they have been shown to capture the impact of various diseases and environmental factors in a single metric^5,6^.

In recent years, advances in the field have led to numerous improvements. First, population-based cohorts used for training have increased from modest sizes to include and combine large cross-sectional studies with thousands of participants^1,2,7,8^. Second, the complexity of models has advanced on two fronts. Machine learning techniques are now used increasingly to develop epigenetic clocks that capture nonlinear and interaction dynamics^9,10^. Furthermore, ‘composite’ or ‘second-generation’ clocks have been directly trained on quantitative markers tracking health and longevity. As a result, these clocks have increased associations with a number of diseases as well as overall mortality^11–13^.

While more sophisticated algorithms and larger cohort sizes have improved the accuracy of epigenetic clocks in predicting chronological age, they do so at the cost of not fully capturing biological information^7^. On the other hand, integration of physiological parameters, such as inflammatory markers, into epigenetic clocks shifted their focus from epigenetic aging toward the prediction of age-related diseases.

Last, a common criticism of most commonly applied epigenetic clocks is that the statistical learning approaches used to predict methylation patterns do not necessarily reflect underlying molecular processes. This limitation complicates the interpretation of their biological underpinnings and has hindered progress in the study of causal mechanisms of biological aging^6,14,15^.

In this study, we systematically identify and evaluate design issues with current epigenetic predictors of chronological age. We show that the amount of biological variability captured by these models decreases as the size of cohorts increases. Further, we highlight how confounding epigenetic processes, such as global change in methylation, can substantially bias predictions of age acceleration.

To overcome these technical limitations and the lack of biological interpretation, we developed a model of epigenetic age acceleration based on a mathematical representation of the cellular dynamics of methylation change^16,17^. While this model does not aim to study the causal role of epigenetic changes in aging, it allows for mechanistic interpretation of methylation change at CpG sites. Its application predicts two distinct processes that modify the natural progression of epigenetic change over the human lifespan: epigenetic age acceleration and bias (global change in methylation levels). We provide an efficient method to infer these parameters for each individual using blood-based methylome-wide array data from the Generation Scotland (GS) cohort and develop a novel batch-correction algorithm to enhance transferability of our model to other cohorts. Further, we develop an open-source online platform that implements our algorithm to facilitate inference in external cohorts (available at https://probage.streamlit.app/).

We also describe associations between our acceleration and bias parameters with lifestyle factors, prevalent disease outcomes and risk of all-cause mortality. We uncover methylation bias as a significant confounding factor in the inference of epigenetic age acceleration in previous clocks. Further, a genome-wide association study revealed seven genomic regions significantly associated with epigenetic age acceleration.

## Results

### Limitations of clocks trained on chronological age

We first sought to understand why CpGs that do not correlate with chronological age are included in epigenetic age predictors^14^. Our findings revealed that inclusion of non-age-correlated CpGs (naCpGs, *R*^2^ < 0.1) improves chronological age prediction at the expense of capturing variability from disease-related lifestyle factors, as we demonstrate below. We used the GSset1 cohort (*n* = 4,450, age range 18–94 years; Methods) for the following experiments.

**Fig. 1:**
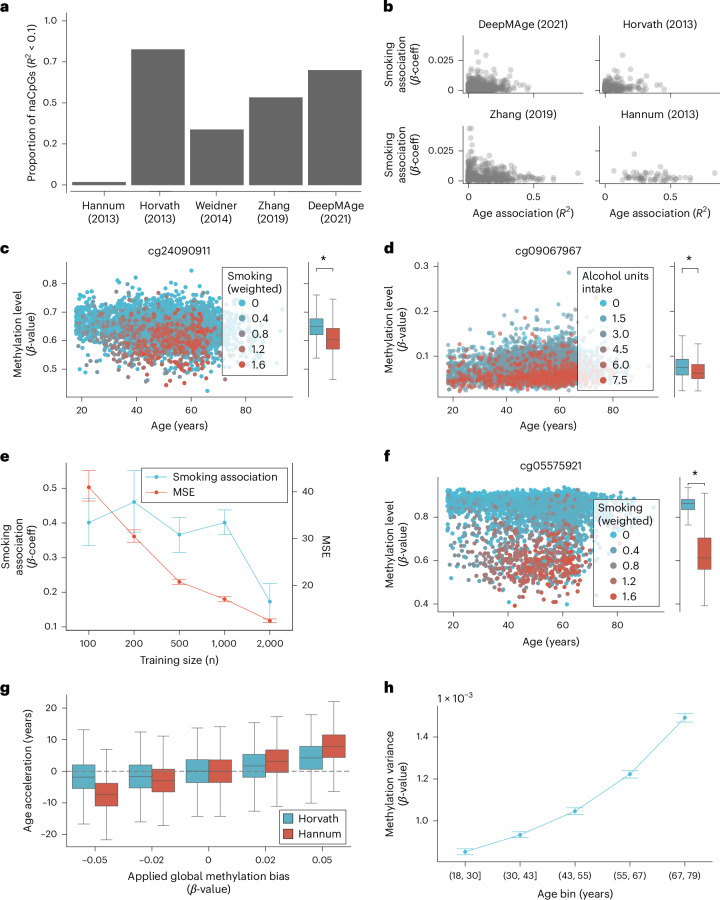
Limitations of current epigenetic predictors. **a**, Proportion of CpGs included in various epigenetic clocks that are not associated with age. For each CpG site included in these epigenetic clocks, we fitted linear regression models of the form: methylation ~ age. naCpGs were defined as sites below an age-association threshold of *R*^2^ = 0.1. **b**, Comparison between the association of methylation levels with smoking and age on each CpG site included in various epigenetic clocks. Each point corresponds to a site included in a clock. Age association displayed as adjusted *R*^2^ from linear regressions for each CpG of the form: methylation ~ age. Smoking association is shown as the absolute value of the β-coefficient from linear regressions for each CpG of the form: methylation ~ smoking + age + sex (Methods). **c**, Methylation level versus age for a single CpG included in the first-generation epigenetic clock of Zhang et al.^7^. Each point represents an individual in the GSset1 (*n* = 4,450) and is colored by their level of smoking (defined in Methods). The CpG displays a negligible association between methylation and age, but strong association between methylation and smoking status. Marginal boxplot shows the median and exclusive interquartile range of methylation levels in heavy smokers (red) and nonheavy smokers (blue) (Methods). The asterisk shows the statistical significance of the difference in methylation levels between these two groups, assessed using an independent two-sample two-sided *t*-test, *P* < 0.001. **d**, Methylation level versus age for a single CpG included in the nonlinear epigenetic clock DeepMAge. Each point represents an individual in the GSset1 (*n* = 4,450) and is colored by their level of alcohol intake (defined in Methods). The CpG displays a negligible association between methylation and age, but strong association between methylation and alcohol intake status. Marginal boxplot shows the median and exclusive interquartile range of methylation levels in individuals with heavy alcohol consumption and low consumption (Methods). Statistical significance of the difference in methylation levels between these two groups was assessed using an independent two-sample two-sided *t*-test, *P* = 0.004. **e**, Acceleration obtained from bootstrapped LASSO linear regressions trained on chronological age for a range of training cohort sizes. Training cohorts were randomly sampled from the GS_set1_ dataset and epigenetic age was predicted for all models on a shared random test set (*n* = 2,000). For each cohort size, the blue line with units on the left *y* axis shows the average and 95% confidence interval of the β-coefficient between epigenetic age accelerations and smoking levels (Methods). For the same cohorts, the red line with units on the right *y* axis shows the average and 95% confidence interval of the mean squared error in the prediction of chronological age. **f**, Methylation level versus age for a single CpG included in all LASSO models of chronological age trained with 2,000 individuals. Each point represents an individual and is colored by their level of smoking (defined in Methods). The CpG displays a negligible association between methylation and age, but strong association between methylation and smoking status. Marginal boxplot shows the median and exclusive interquartile range of methylation levels in heavy smokers and nonheavy smokers (Methods). Statistical significance of the difference in methylation levels between these two groups was assessed using an independent two-sample two-sided *t*-test, *P* < 0.001. **g**, Impact of global offsets on the inferred accelerations using Horvath and Hannum clocks for individuals in GSset1 (*n* = 4,450). Global offsets in methylation were increasingly applied to all sites in GS_set1_ and accelerations were computed as the residual from the predicted age to the chronological age of individuals. Further, accelerations have been shifted for each clock so that the predictions with zero offset are centered at 0. Boxplots show the median and exclusive interquartile range. **h**, Increase of variance of methylation levels across age bins in all age-related CpGs (*R*^2^ > 0.1, *n* = 13,875). Participants in GS_set1_ were used to assess the variance in methylation levels across age bins. Age bins were equally distributed in age range (12.4 years) and samples (*n* = 294), to limit under or over estimation of variance across bins. For each bin we show the mean variance in methylation levels across all sites with error bars showing 95% confidence intervals. The *y* axis values are reported in a scientific scale (1 × 10^−3^).

**Extended Data Fig. 1:**
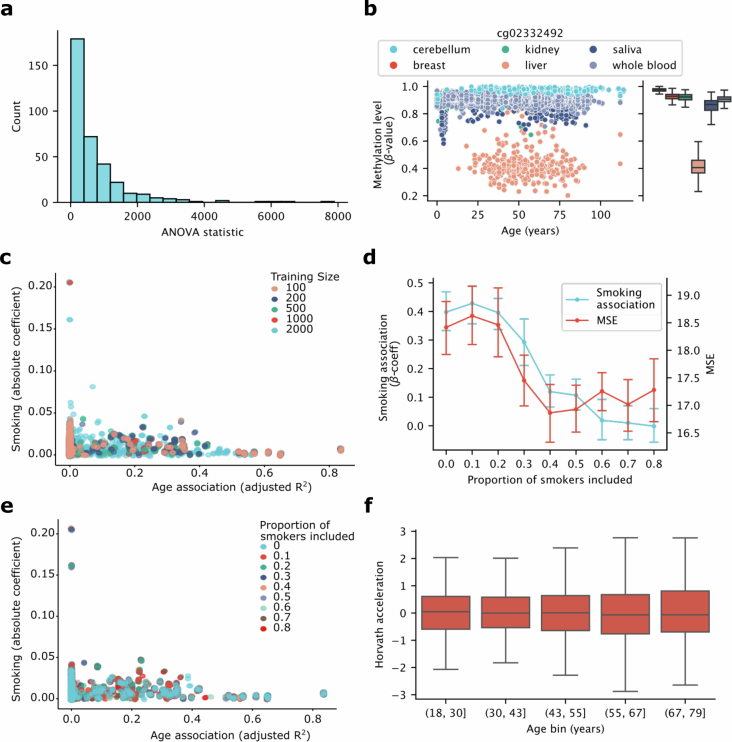
Analysis of current limitations in epigenetic clocks. **a**. Distribution of ANOVA statistics between six example tissues, for each site included in the Horvath clock when calculated in EWAS Datahub dataset. **b**. Methylation level vs age for a single CpG included in the first-generation epigenetic clock of Horvath. Each point is a cell and is colored by the tissue type it was taken from (all six tissue types were included in the training dataset of the original Horvath clock). The CpG displays negligible association between methylation and age, but strong association between tissue type, in this case liver. Marginal boxplot shows the median and exclusive interquartile range of methylation levels in six different tissues. **c**. Comparison between the association of methylation levels with smoking and age on each CpG site included in a training size bootstrap experiment. Each point corresponds to a site included in a clock, colored by the size of the dataset used to train the epigenetic predictor and sized proportionally to the smoking coefficient value. Age association displayed as adjusted R^2^ from linear regressions for each CpG of the form: methylation ~ age. Smoking association shown as the absolute value of the coefficient of smoking (dichotomized as a weighted smoking value of greater than 0.25) from regressions for each CpG of the form: methylation ~ smoking + age + sex. Weighted smoking is defined as log(1 + pack years)/exp(ever smoke). Points are displayed with a random jitter to avoid overlap. **d**. Acceleration obtained from bootstrapped lasso linear regressions trained on chronological age trained on datasets of increasing proportions of smokers and a fixed size (n=700). Training cohorts were randomly sampled from the GS_set1_ dataset. The results were computed on random test sets (n=2000). The red line, associated to the left y-axis, shows the average, and 95% confidence interval, association between the predicted age accelerations with smoking, given training cohort size. The blue line, associated to the right y-axis, shows the average, and 95% confidence interval, mean squared error in the prediction of chronological age. **e**. Comparison between the association of methylation levels with smoking and age on each CpG site included in a bootstrap experiment of the effect of the proportion of smokers. Each point corresponds to a site included in a clock, colored by the size of the dataset used to train the epigenetic predictor and sized proportionally to the smoking coefficient value. Age association displayed as adjusted R^2^ from linear regressions for each CpG of the form: methylation ~ age. Smoking association shown as the absolute value of the coefficient of smoking (dichotomized as a weighted smoking value of greater than 0.25) from regressions for each CpG of the form: methylation ~ smoking + age + sex. Weighted smoking is defined as log(1 + pack years)/exp(ever smoke). Points are displayed with a random jitter to avoid overlap. **f**. Increase of variance in Horvath clock acceleration with age. Box plots show the median and exclusive interquartile range of the accelerations. Age bins were equally distributed in age range (12.4 years) and samples (n=294), to limit under or over estimation of variance across bins.

First, we showed the extent to which naCpGs are incorporated in commonly used clocks trained on chronological age (Fig. 1a). We excluded composite epigenetic clocks from our analysis because they are expected to include naCpGs that predict phenotypes other than chronological age^11,12^. Note that the high number of naCpG sites present in the multi-tissue predictors, Horvath^3^ and Skin and Blood^5^, might be explained in part by the presence of tissue-specific sites. For example, we used data from 3,717 samples across six tissues, included in the EWAS Datahub^18^ and found that methylation levels in most naCpGs included in Horvath’s epigenetic clock (*n* = 288) were highly associated with tissue of origin (*P* < 1 × 10^−5^; Methods and Extended Data Fig. 1a,b). Second, we plotted the age association of all clock CpGs against their association with smoking, which has widespread associations with blood-based DNAm^19,20^ (Fig. 1b and Methods). This revealed that some CpGs with a very low age association displayed a high association with smoking, possibly explaining their inclusion in the clocks. In Fig. 1c, we showed an example of a smoking-correlated CpG site included in the Zhang et al. clock^7^. In this site (cg24090911, *AHRR*), methylation levels displayed a more pronounced correlation coefficient with smoking levels (*R*^2^ = 0.1, *P* < 0.001) than age (*R*^2^ = 0.02, *P* < 0.001). Further global methylation levels of *AHRR*, the gene associated with cg24090911, have been well described to track long term smoking behavior^21^. A further independent two-sample *t*-test comparing the methylation levels in this site between heavy smokers and nonheavy smokers (Methods) revealed a significant association, *t*(4,448) = −20 and *P* < 0.001. Smoking being the predominant source of variation suggests that inclusion of this site in the epigenetic clock reduced the impact of biological variability associated with smoking to increase the accuracy of chronological age predictions. Similarly, in Fig. 1d, we showed a site (cg09067967) included in the DeepMAge clock that shows higher correlation with alcohol consumption (*R*^2^ = 0.04, *P* < 0.001) than age (*R*^2^ = 0.01, *P* < 0.001). An independent two-sample *t*-test comparing the methylation levels in this site between heavy drinkers and nonheavy drinkers (Methods) revealed a significant association, *t*(4,448) = −2 and *P* = 0.004. As DeepMAge is a nonlinear neural network predictor and alcohol consumption is a correlate of epigenetic age predictors^9,19^, this suggests that incorporation of naCpGs persists even when more complex algorithms are applied.

To further investigate the incorporation of naCpGs in linear predictors of chronological age, we investigated how an increase in cohort size impacts age prediction accuracy and association with tobacco smoking, a lifestyle factor associated with poor health outcomes^22^ (Fig. 1e,f). We used a subset of the GS cohort (GS_set1_; *n* = 4,450 with ages ranging 18–93 and blood-based Illumina EPIC array DNAm; Methods and Supplementary Methods) and bootstrapping techniques to train LASSO regression models on training sets of increasing sizes (Methods). We found that an increased size of the training cohort results in more accurate chronological age prediction, as previously reported^7^ (Fig. 1e). In contrast, the strength of association between smoking and epigenetic age acceleration estimated by the bootstrapped models showed a sharp decline for training sets exceeding 1,000 individuals. This drop in association correlates with an increasing fraction of naCpG sites being considered, some of which correlate strongly with smoking (Extended Data Fig. 1c). In Fig. 1f, we showed that naCpG (cg05575921, AHRR) is strongly associated with smoking (*R*^2^ = 0.59, *P* < 0.001), which becomes incorporated in all models trained with 2,000 individuals. These results suggest that as the size of the training set increases, the model is able to observe larger biological differences across participants, such as larger differences in smoking habits, and favors incorporation of naCpGs during training to fine-tune its predictions for subsets of people. To further test this hypothesis, we tested the effect of increasing the prevalence of smokers in training cohorts of fixed size (*n* = 700; Methods). This test produced analogous results: the strength of association between smoking and epigenetic age acceleration decreases as the prevalence of smokers increases (Extended Data Fig. 1d,e). Similarly, the naCpG illustrated in Fig. 1f is incorporated in every clock trained with more than 30% of smokers. Again, these results suggest that as the model was exposed to larger variations in smoking habits during training, it favored incorporation of naCpG sites associated with smoking.

Taken together, these analyses showed that inclusion of naCpGs acting as indicator variables for other lifestyle features, for example smoking, allows the model to fine-tune its predictions for subsets of people, improving the predictive performance of epigenetic clocks for chronological age at the expense of capturing the epigenetic variability between individuals.

We next considered global biases in methylation levels. These may occur due to incomplete bisulfite conversion or as the result of an underlying biological process. The imbalanced contribution of hyper- and hypomethylating CpGs in epigenetic predictors results in over- or underestimation of the inferred acceleration in the presence of a global bias (Supplementary Methods 2.1). To test this hypothesis, we offset GS_set1_ global methylation levels both positively and negatively and applied several published epigenetic clocks to the modified data (Fig. 1g). This showed that, while the robustness against global methylation biases varied across clocks, it was susceptible to shifts in acceleration predictions.

Finally, acceleration is commonly defined, uniformly for all ages, as the residual between clock predictions and chronological age. We estimated the time evolution of the variance of methylation levels, in all CpG sites correlated with age (*R*^2^ > 0.1), and observed an overall increase with age (Fig. 1h and Methods), as seen in other studies^23^. While drifting methylation levels in CpG sites may be part of the natural progression of aging patterns in methylation, it challenges the fundamental assumption of homoscedasticity in linear epigenetic clocks. This natural drift in methylation levels at a later age in human life, therefore, results in artificially inflated epigenetic age accelerations in first-generation clocks that may be confounded with biological variability (Extended Data Fig. 1f).

**Fig. 2:**
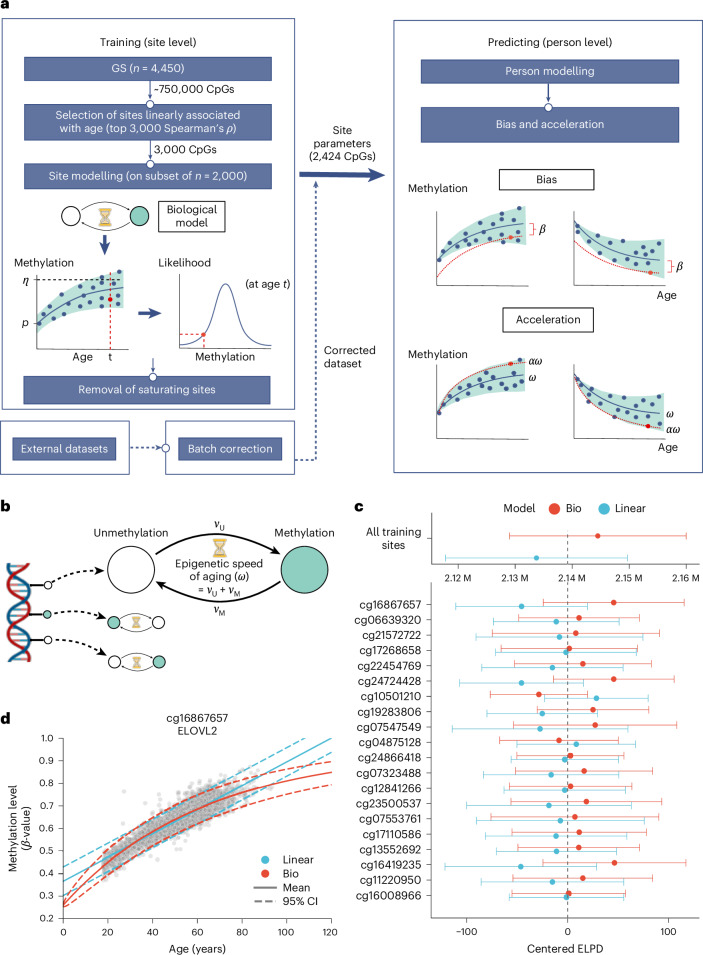
Workflow and modeling methylation on CpG sites. **a**, Overview of the study workflow showing how site-specific parameters are derived across the cohort and then used to calculate bias and acceleration values for each individual. There is a separate pipeline for the external datasets which does not involve training. Created with BioRender.com. **b**, Schematic showing the biological model’s underlying stochastic process. For a single cell, multiple CpG sites can over time either methylate or demethylate with rates *V*_U_ and *V*_M_, respectively. Created with BioRender.com. **c**, Comparison between the linear and biological model in CpG sites. Sites are compared using the expected log-predictive density (ELPD), approximated using Pareto-smoothed importance sampling. This measure penalizes the model for a higher number of parameters and gives a higher value for a model that better explains the data. For each comparison we show the mean and 2 × s.d. of the ELPD. Top plot shows the model comparison across all 1,024 sites used for model training. The bottom plot shows the model comparison for the 25 sites showing the highest Spearman correlation square. In the bottom plot, the ELPD of each comparison has been centered at zero to facilitate the display of many sites. **d**, Predicted dynamics of methylation levels in CpG 16867657 by the biological and linear model. Gray dots show the methylation and age of individuals in the GS_set1_ cohort. Red lines show the biological model’s mean and 95% prediction interval. Blue lines show the linear model’s mean and 95% prediction interval.

These limitations of epigenetic age predictors (summarized in Fig. 1) and the absence of a mechanistic biological foundation necessary for interpreting their outcomes motivated us to devise an alternative approach for measuring epigenetic age acceleration (Fig. 2a).

### A biological model of methylation changes

Mechanism-based mathematical models offer a tangible interpretation of biological processes driving methylation pattern change over time. At the single cell level, methylation changes at each genomic locus can be interpreted as transitions between the unmethylated and methylated state, with rates *V*_U_ and *V*_M_, respectively (Fig. 2a,b). This parsimonious model was first proposed in 1990 to study the clonal inheritance of CpG patterns^16^. Here we extend this model in the context of time-dependent dynamics of methylation changes to capture the effect of altered rates of epigenetic aging.

The dynamics of methylation changes over time at each site can be described with interpretable parameters. A coarse interpretation of the model dynamics is that in each CpG site, methylation levels transition from methylation level $p$ to $\eta$ at speed $\omega$ (Methods and Supplementary Methods for a complete description of the model). More precisely, parameters $p$ and $\eta$ determine the initial and terminal proportions of methylated cells and hence also reflect the directionality of methylation change with age at each locus. Notice that these methylation states are described by the mathematical model rather than a particular biologically marked timepoint. That is, this model assumes that, in each genomic loci, methylation patterns transition from an initial state $p$, which may have occurred before birth to a steady state $\eta$ that may not be achieved during the human lifespan. Parameter $\omega$, on the other hand, determines the total rate of transitions between methylation states. This corresponds to the speed at which methylation levels transition from the initial to the terminal state at a single CpG site (Fig. 2a,b and Methods). Further parameters are involved in the evolution of variance over time (Methods).

This formulation, therefore, allows derivation of a biologically informed notion of epigenetic age acceleration of an individual. The dynamics associated with an acceleration, *α* simply correspond to a uniform change, across all sites, in the speed of methylation change, $\omega$. That is, methylation levels in an individual with an epigenetic acceleration *α* will transition in each site from $p$ to $\eta$ at a modified speed $\alpha \times \omega$ (Fig. 2a and Methods). Further, this model allows inference of global hypo- or hypermethylating changes altering the whole methylome of an individual, termed bias or *β* (Fig. 2a and Methods).

To facilitate the interpretation of the model’s parameters we have developed a web application that allows interactive exploration of their role at https://probage.streamlit.app. Further, the linear approximation of this biological model provides a familiar interpretation of epigenetic acceleration and bias. An increase *α* in the speed of aging at a single CpG site corresponds to a proportional increase of the slope in methylation trajectories. Similarly, bias corresponds to a uniform shift of the intercepts (Supplementary Methods).

**Extended Data Fig. 2:**
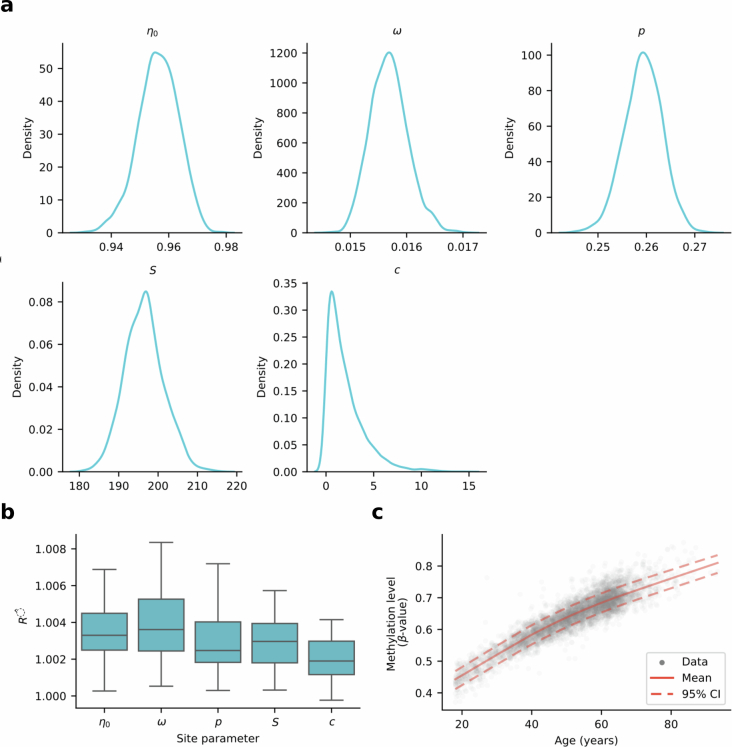
Site modelling. **a**. Posterior distribution of the 5 site parameters for an example CpG site (ELOVL2). Each X axis shows the density of the distribution **b**. Scatter plot representing the Posterior Predictive Check (PPC) of inferred site parameters. Gray points represent methylation values sampled from the posterior shown in Extended Data Fig. 2a. X axis shows the methylation value expressed in beta values, Y axis is age in years. Red lines show the biological model’s mean and 95% confidence interval predictions. **c**. Boxplot showing the median and exclusive interquartile range of convergence diagnostics R-hats for 5 site parameters for all sites used after the selection of not saturating sites.

We used the GS_set1_ cohort (*n* = 4,450, age range 18 to 94 years; Methods) to fit our biological model and infer the parameters for each CpG site with a high age correlation (Spearman correlation squared *ρ*^2^ > 0.2, *n* = 1,723). For visual inspection of parameter stability, we have included an example of the posterior distributions of ELOVL2 site parameters (Extended Data Fig. 2a) and generated posterior predictive samples from the model (Extended Data Fig. 2b). We have also included the distribution of convergence diagnostics R-hats for all sites used in the later evaluation (Extended Data Fig. 2c).

To compare the fit of our approach with the linear model, we used Bayesian techniques to approximate the predictive power, expected log-predictive density, of both models in CpG sites (Methods). Our proposed biological model outperforms linear modeling when comparing each considered CpG site individually in 97% of the cases (Fig. 2c,d) and globally across all CpG sites considered together (Fig. 2c). As the biological model predicts nonlinear dynamics, it is capable of capturing complex changes in both mean and variance that linear models fail to explain (Fig. 2d).

**Fig. 3:**
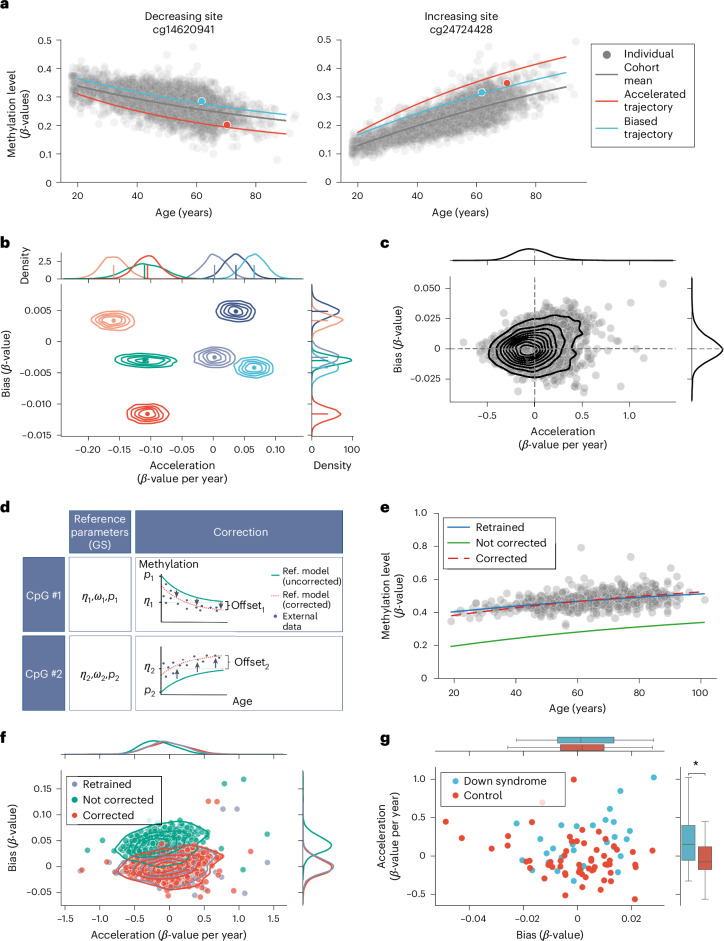
Acceleration and bias. **a**, Differences between acceleration and bias in model predictions. Predicted evolution of both an accelerated but not biased (red) individual, and of a biased but not accelerated individual (blue) in GS_set1_. The trajectories for the same individuals are shown for both sites with decreasing and increasing methylation levels with time in separate panels. Acceleration increases the *slope* of the predicted evolution of methylation levels, whereas bias globally offsets regardless of the direction of the slope. In gray we show the methylation and age values for the rest of individuals in GS_set1_ and the predicted mean evolution of methylation levels. **b**, Joint posterior distribution of the acceleration and bias for six individuals in GS_set1_ (*n* = 4,450). Each individual is identified by a unique color. The maximum a posteriori (MAP) value is also highlighted with a point. The marginal posterior distribution of the acceleration and bias are shown in marginal plots for each individual with an additional line showing the MAP value. **c**, Inference results of acceleration and bias for all individuals in the GS_set1_ cohort (*n* = 4,450). Marginal plots show the Kernel density estimate (KDE) of the distribution of inferred MAP values of acceleration and bias for all individuals in GS_set1_. **d**, Schematic illustrating how batch correction is applied on each site in an external dataset. An offset is inferred for each CpG site as the uniform shift in the predicted dynamics of our biological model that maximizes the probability of observing all individuals in the external dataset. Created with BioRender.com. **e**, Application of batch correction on a single CpG site (cg00048759) in the Hannum dataset. Green line shows the mean methylation trajectory displayed in the GS_set1._. Blue line shows the trajectory inferred from a new model trained on the Hannum dataset. The red dashed line shows the batch-corrected trajectory from GS_set1_ optimized to fit the Hannum dataset. **f**, Effect of batch correction on the inference of acceleration and bias values. Dots show the acceleration and bias inferred by the retrained (blue), not corrected (green) and corrected (red) models. Marginal plots show the KDE of each parameter’s distributions. **g**, Distribution of acceleration and bias inferred in a cohort of individuals with Down syndrome (control (*n* = 58) and disease (*n* = 29), see Methods for details). Our model was batch-corrected using the control group, and acceleration and bias were computed for both control and disease groups. Each dot represents an individual in the external cohort and is colored according to their disease status. Marginal boxplots show the median and exclusive interquartile range of the acceleration and bias, with a separate boxplot for control and disease group. Statistical significance of the difference in methylation levels between these two groups was assessed using an independent two-sample two-sided *t*-test, *P* = 0.005.

We then leveraged the inferred parameters, describing the time evolution of methylation levels in each site, to add a second modeling layer that allows assessing the probability of observing an individual’s methylation profile across all described CpG sites (Fig. 2a). Further, we implemented this layer in a Bayesian framework that allowed us to associate individual-specific deviations from the expected methylation patterns with differences in epigenetic age acceleration and bias (Fig. 3a,b and Methods). Critical for the success of this model is the capacity to disentangle the effects of both. The effects of acceleration and bias in methylation patterns can, however, be separated thanks to the CpG specific directionality of methylation patterns of age, that is, in each age-related CpG site methylation changes occur in a single direction (either gain or loss of methylation with time). Bias reflects global changes in methylation and is therefore independent of the directionality of methylation levels in age-related CpG sites, whereas acceleration is linked to the specific directionality of methylation changes at each genomic locus (that is it results in either faster methylation or faster demethylation, depending on the site) (Fig. 3a). Further, Bayesian analysis of the posterior distribution of the inferred acceleration and bias of a random subset of individuals suggested that our framework is able to disentangle these parameters (Fig. 3b).

Next, we performed stability analysis to assess the effect using an increasing number of CpG sites in the inference of acceleration and bias. We used increasing numbers of CpG sites (sorted by their Spearman correlation squared with age, *ρ*^2^) to infer acceleration and bias, and showed that model predictions become stable when using more than 512 sites (Extended Data Fig. 2a,b and Methods). The reported acceleration and bias used in all further analyses were those obtained from using 512 sites.

**Extended Data Fig. 3:**
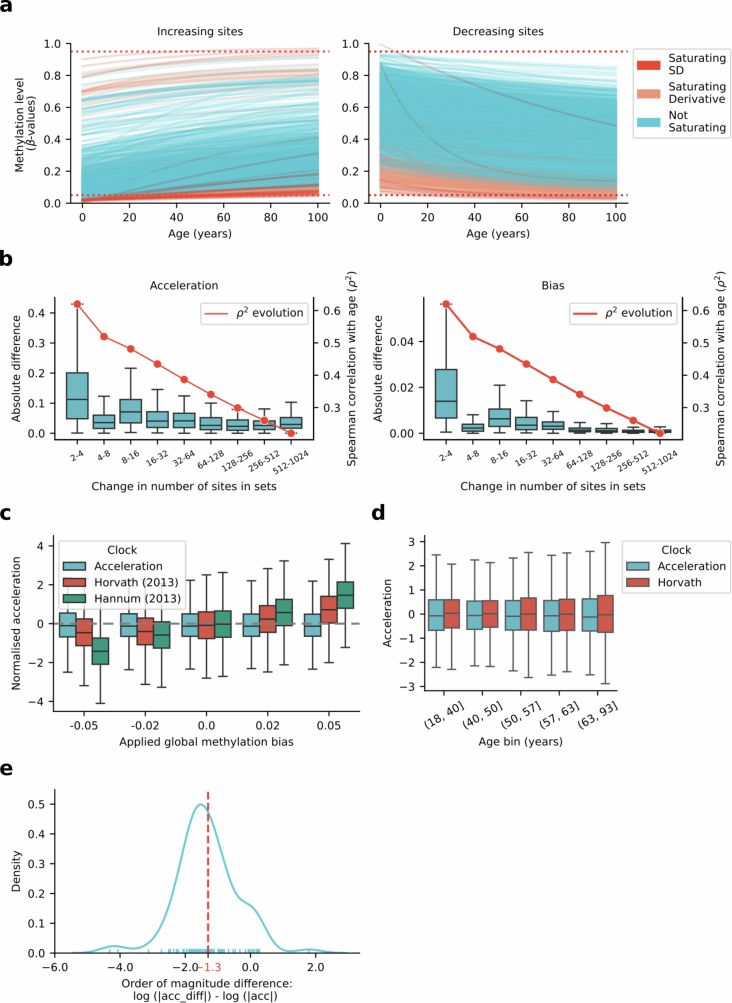
Tuning and testing the stability of epigenetic age acceleration and bias. **a**. Filter of saturating sites showing increasing (left panel) and decreasing (right panel) methylation trends. Each line shows the predicted time evolution of the mean methylation level of a single CpG site. Red lines represent sites saturating based on the mean value at the age of 90, orange lines represent sites saturating based on the derivative value of our model prediction at the age of 90. Blue lines indicate sites that have not been filtered and are taken forward for acceleration and bias fitting. Horizontal dashed lines at values 0.05 and 0.95 are included, to show the threshold for the mean saturation filter. **b**. Effect of increasing the number of CpG sites on the inferred acceleration (left) and bias (right) for all participants in the GS_set1_ cohort. Acceleration and bias were fitted using exponentially increasing numbers of CpG sites decreasingly ordered in terms of the coefficient of determination, R^2^, between the methylation levels and age of participants in each site. Boxplots show mean and exclusive interquartile range of the absolute difference in consecutive inferences of acceleration and bias. Additionally, a the lowest R^2^ value included in each model is shown in red for each iteration, with values shown on the second Y-axis **c**. Impact of different global offsets, affecting all CpG sites uniformly, on the accelerations inferred by our model and Horvath’s epigenetic predictor. Accelerations are computed without applying the batch correction. Box plots show the median and exclusive interquartile range of the accelerations inferred for all individuals in the GS_set1_. Acceleration values are scaled by the standard deviation observed in GS_set1_ to facilitate a direct comparison. In the biological model, bias absorbs the effect of global changes in methylation and results in a stable acceleration measure. **d**. Change of variance of our and Horvath acceleration inferred for all individuals in the GS_set1_, across age bins. Box plots show the median and exclusive interquartile range of the accelerations. Age bins were equally distributed in age range (12.4 years) and samples (n=294), to limit under or over estimation of variance across bins. **e**. Distribution of order of magnitude difference between the predictions of the masked model on the older population (>75) and the predictions of the full model (trained using all participants). The X axis shows the resulting logarithmic difference (see Methods). The Y axis shows the density of a KDE curve. The dotted red line and tick show the mean order difference.

The distributions of acceleration and bias inferred from all individuals in the GS_set1_ cohort is shown in Fig. 3c. This highlights that individuals can exhibit increased acceleration or bias, or a combination of both. We then showed that the inferred accelerations are robust against global changes in methylation (Extended Data Fig. 3c) and that their variance increases with age, albeit less pronounced than Horvath’s epigenetic age accelerations (Extended Data Fig. 3d). Given that our model takes into account the increase in variance of methylation dynamics with age, this suggests that the increase in variance of the inferred accelerations with age relates to an increase in biological variability rather than the dispersion of stochastic patterns of methylation changes^17^.

Further, to assess the impact of survivorship bias in our predictions of epigenetic age acceleration, we trained a new model removing all people over the age of 75 in the training dataset (below life expectancy in Scotland for both males and females). This model should therefore be relatively unaffected by survivorship bias. We compared the predictions of this masked model on the older population (>75) with the predictions of the full model (trained using all participants). Analysis of the distribution of order of magnitude of differences between both model predictions (Methods) revealed that the potential change in acceleration prediction caused by survivorship bias is consistently below the order of magnitude of the predicted acceleration for each individual (Extended Data Fig. 3e; mean = −1.3, s.d. = 1.13). For example, for an older individual with an acceleration of 0.1, survivorship bias could only account for a change in acceleration of 0.01.

### Batch correction

**Extended Data Fig. 4:**
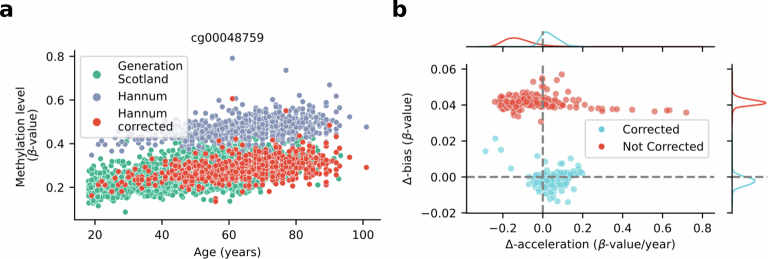
Batch correction. **a**. Visual interpretation of the effect of batch correction between GS_set1_ and the Hannum external dataset on a single CpG site. Blue dots show the methylation levels and ages of individuals in the reference GS_set1_ cohort are plotted in blue. Red dots show the methylation levels and ages of individuals in the external Hannum cohort. Blue dots show the optimal offset inferred by our batch-correction algorithm. **b**. Effect of batch correction on the inference of acceleration and bias values in the Hannum dataset. Dots show the difference from the acceleration and bias using the corrected (blue) and not corrected (red) models to that inferred using the retrained model. Marginal plots show the KDE of each parameter’s distributions.

We designed a batch-correction algorithm to allow comparisons across cohorts. To enable transferability of our model to an external cohort, without the need to retrain, we designed an inference framework to fine-tune a site-specific offset applied to the reference model parameters’ that maximizes the overlap in methylation dynamics across datasets (Fig. 3d and Methods). We tested the validity of our batch-correction algorithm on the Hannum et al. cohort^4^. In Fig. 3e (and Extended Data Fig. 4a) we provide a visual comparison of the effect of our batch-correction algorithm on the site showing the largest offset. We showed minimal differences in predictions between our batch-corrected model and a fully retrained model on the external dataset (Fig. 3f and Extended Data Fig. 4b).

To further validate our batch-correction algorithm, we investigated its applicability to datasets with limited sample sizes. We used publicly available data of a Down syndrome cohort (*n* = 58 control, *n* = 29 disease)^24^. As expected, despite the dataset’s small number of participants, Down syndrome was associated with independent two-sample *t*-test, *t*(85) = 3.1 and *P* = 0.002 (see Fig. 3g and Methods), as previously described^24^.

### Acceleration is associated with lifestyle factors and health outcomes

Next, we tested the association of age acceleration and bias with disease phenotypes and genetic variants. The following analyses were conducted on the combined subsets of GS (*n* = 15,900): GS_set1_ (*n* = 4,450) the batch-corrected subset GS_set2_, (*n* = 2,578, unrelated to each other and to GS_set1_, and processed in separate experimental batches) and the batch-corrected subset GS_set3_ (*n* = 8,872, processed in separate experimental batches).

**Fig. 4:**
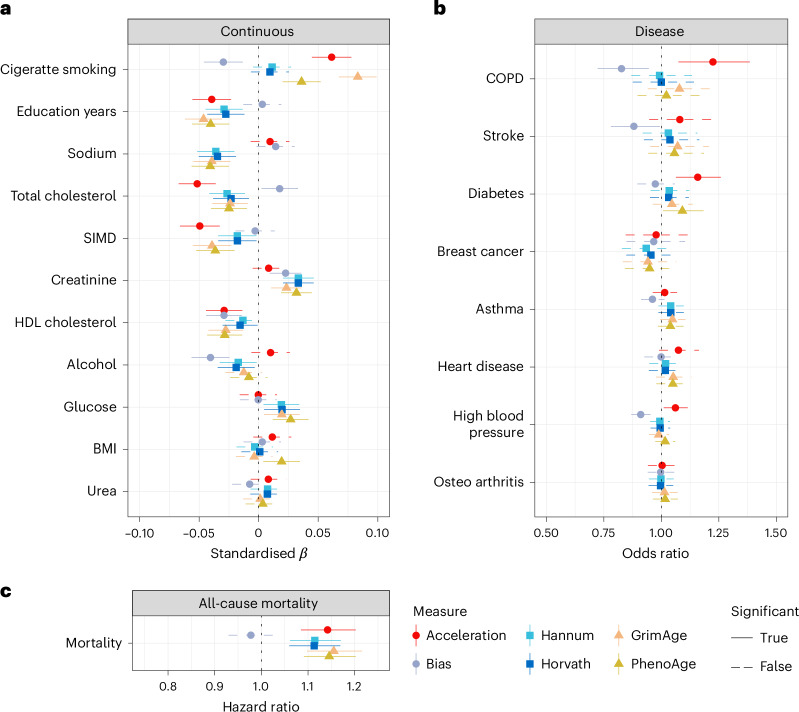
Phenotypic association studies. **a**, Associations between acceleration, bias and continuous traits. Forest plot of associations between continuous phenotypes and age acceleration (red), bias (purple), Hannum (light blue), Horvath (dark blue), GrimAge (orange), PhenoAge (yellow). Significant associations are defined using FDR-adjusted two-tailed *P* < 0.05. Dots denote standardized beta value and horizontal lines represent 95% CIs estimated from linear regression models of the form: phenotype ~ acceleration + bias + age + sex. SIMD (Scottish Index of Multiple Deprivation). **b**, Associations between acceleration, bias and disease traits. Forest plot of associations between disease phenotypes and age acceleration (red), bias (purple), Hannum (light blue), Horvath (dark blue), GrimAge (orange), PhenoAge (yellow). Significant associations are defined using FDR-adjusted two-tailed *P* < 0.05. Dots denote odds ratio and horizontal lines represent with 95% CIs estimated from logistic regression models of the form: disease ~ acceleration + bias + age + sex. COPD (chronic obstructive pulmonary disease). **c**, Associations between acceleration, bias and all-cause mortality. Forest plot of associations between all-cause mortality and age acceleration (red), bias (purple), Hannum (light blue), Horvath (dark blue), GrimAge (orange), PhenoAge (yellow). Significant associations are defined using FDR-adjusted two-tailed *P* < 0.05. Dots denote hazard ratio and horizontal lines represent 95% CIs estimated from a Cox proportional hazards model of the form hazard ~ acceleration + bias + age + sex. The associations reflect an elevation of one standard deviation in the relevant measure of biological aging.

We performed linear regression analyses to quantify associations between our two measurements (acceleration and bias) and disease phenotypes while controlling for age and sex (Fig. 4a–c and Supplementary Tables 1–3). This revealed significant (false discovery rate (FDR)-corrected *P* < 0.05) associations between age acceleration and five physiological traits and lifestyle factors. Increased acceleration was associated with higher levels of tobacco smoking, greater deprivation, lower levels of total cholesterol and high-density lipoprotein (HDL) cholesterol. Increased acceleration was also associated with an increased risk of several self-reported prevalent disease outcomes, namely diabetes, chronic obstructive pulmonary diseases (COPD) and high blood pressure. Further, a Cox proportional hazards regression analysis showed an association between acceleration and all-cause mortality (Fig. 4c; HR = 1.14 per s.d. increase of acceleration (95% confidence interval 1.085–1.202), *P* < 0.05). Similarly, increased bias was associated with four physiological traits, namely lower levels of tobacco smoking, lower levels of alcohol consumption, lower levels of HDL cholesterol and higher levels of creatinine. Moreover, increased bias correlated with lower incidences of high blood pressure and COPD.

We conducted a comprehensive performance comparison between our model and the four most widely used epigenetic clocks: Horvath, Hannum (first generation), PhenoAge and GrimAge (second generation) (Fig. 4a–c). This comparison, however, has to be carefully interpreted as our model decouples two distinct phenomena, acceleration and bias, which would have been confounded in a single acceleration metric in previous clocks (Supplementary Information Methods 2.1).

Compared to first-generation clocks, which like our model exclusively depend on methylation data and do not incorporate phenotypic trait information, we observe significant associations between acceleration and well-established age-related modulators that these epigenetic clocks fail to capture. These include smoking, HDL cholesterol, COPD, diabetes and high blood pressure. Furthermore, we find increased associations in total cholesterol, socioeconomic deprivation index (SIMD), body mass index, heart disease and all-cause mortality.

Second-generation clocks, on the other hand, are more difficult to compare as their associations with age-related modulators and traits is highly dependent on the specific phenotypic traits used in the training of these clocks. For example, GrimAge included smoking and all-cause mortality in the training of the model parameters. Nevertheless, our model still outperforms these clocks in finding significant associations for COPD and high blood pressure and shows comparable associations in most other traits (Fig. 4a–c).

Comparing our results to those associations found using first-generation clocks underscores the importance of incorporating global methylation bias as a parameter. In the context of smoking, for example, we found a significant association with both a negative bias and increased acceleration, whereas no association was found with epigenetic age in first-generation clocks. Notice that, as described in Fig. 1g, a global decrease of methylation levels, or negative bias, negatively skews the epigenetic age predictions of the Horvath and Hannum clocks. In these clocks, therefore, a combination of negative bias and increased epigenetic age acceleration skews epigenetic age predictions in opposing ways and results in the absence of any association with smoking. This opposing pattern is also found in other traits such as COPD, total cholesterol and high blood pressure, where associations are either decreased or absent in first-generation clocks.

Confounding acceleration and bias may also explain the spurious correlation linking increased alcohol consumption with younger epigenetic age in first- and second-generation clocks. Our algorithm links increased consumption to both a mild increase in epigenetic age acceleration and a strong negative bias, or global hypomethylation. Notably, global depletion of methylation levels in the context of high alcohol consumption has been previously reported^25^. These opposing dynamics between acceleration and bias are also observed in total cholesterol levels and COPD and high blood pressure incidence resulting in insignificant associations in previous clocks.

These nuanced observations underscore the importance of understanding the biological mechanisms underpinning different methylation clocks and highlight the unique insights our model provides.

### GWAS of acceleration implicates age-related genomic loci

**Fig. 5:**
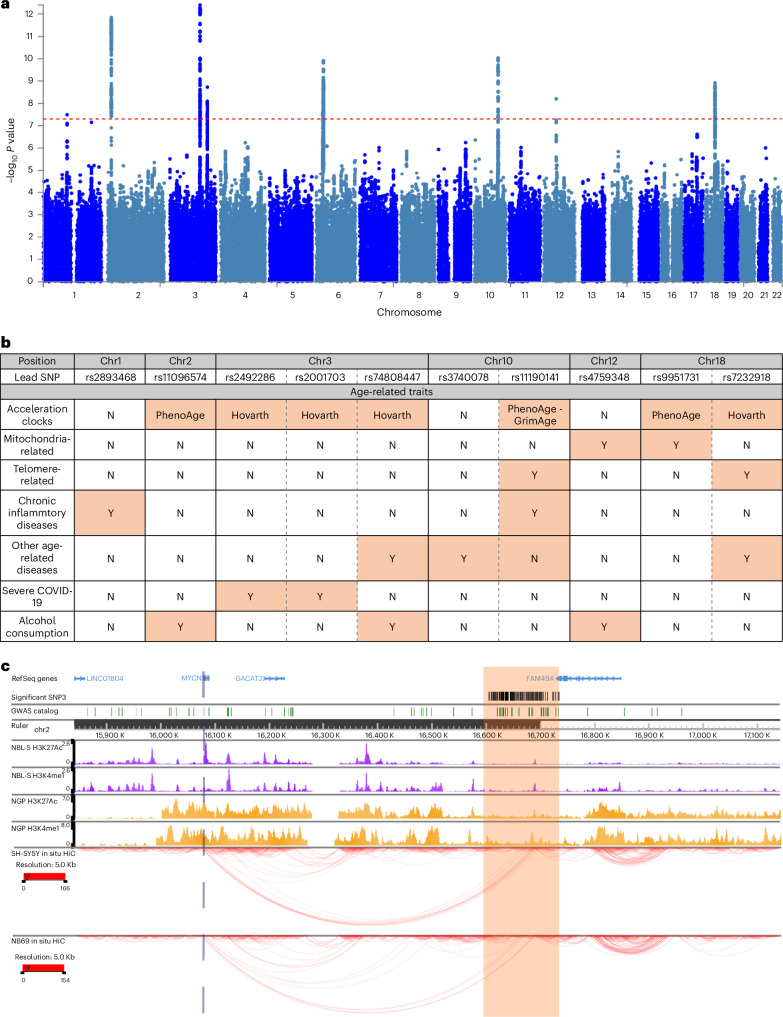
Genome-wide association studies. **a**, Manhattan plot of results from the genome-wide association analysis of age acceleration. Red dotted line indicates the genome-wide significance threshold (*P* < 5 × 10^−8^). **b**, Presence of GWAS catalog trait associations of any SNPs in LD (*R*^2^ > 0.1) with the lead SNP in the six genome-wide significant genomic loci. Traits are grouped into seven categories of interest. **c**, A zoomed-out view of chromatin interactions and chromatin features in and around the genomic risk loci chr2:15841100–17142797 (highlighted in orange). Significant SNPs are shown as red vertical lines. SNPs identified by published GWAS collected in the NHGRI-EBI GWAS catalog are shown as green vertical lines. ChIP-seq for H3K27ac and H3K4me1 in NBL-S cells and NGP cells are shown as purple histograms and orange histograms, respectively. Colored arcs depicting In situ HiC chromatin contacts in SH-SYSY cells and NB69 cells. Demonstrates interaction of the genomic risk loci with the *MYCN* gene locus.

We then conducted a genome-wide association analysis to identify genetic variants that are associated with acceleration. We found 853 single-nucleotide polymorphisms (SNPs) at genome-wide significance (*P* < 5 × 10^−8^) clustered in seven genomic regions on chromosomes 1, 2, 3, 6, 10, 12 and 18 (Fig. 5a). The genomic risk locus situated on chromosome 6 was within the highly polymorphic MHC locus and thus was excluded from downstream analysis. SNPs located in the acceleration- associated genomic risk regions were also associated with age-related traits via look-up analyses in the GWAS catalog^25^ (Fig. 5b). All regions were associated with at least one age-related trait and rs10883360, located in chromosome 10, was associated with three categories, namely telomere size, chronic inflammatory diseases and other age-related diseases. Seven out of the 11 reported independent lead SNPs demonstrate linkage disequilibrium with SNPs associated with age acceleration as measured by other aging clocks^26^ (Horvath, PhenoAge or GrimAge) and SNPs within the chromosome 10 region (chr10: 101271789–101589328) were associated with telomere length.

**Extended Data Fig. 5:**
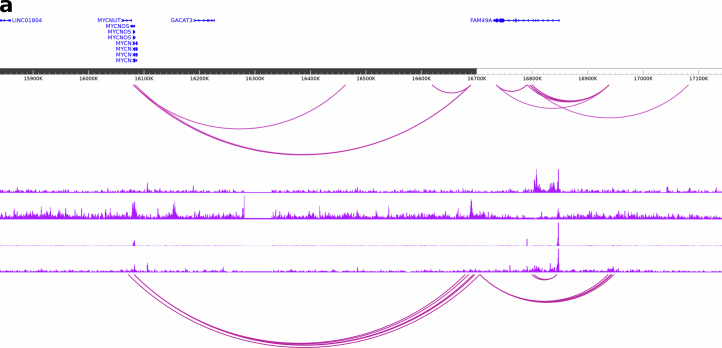
Genome-wide association. **a**. A WashU genome browser showing genetic and epigenetic information within and around the genomic risk loci chr2:15841100-17142797 (highlighted in orange). Blue histograms indicate PBMC ChIP-seq data, while top purple arcs depict SMC1 ChIA-PET data in T-ALL Jurkat cells, and bottom purple arcs represent promoter capture HiC data in PBMC cells.

We then performed downstream functional analysis to link SNPs with genes by overlapping the genomic regions with peripheral blood mononuclear cell (PBMC) promoter capture HiC data^27^ and PBMC ChIP-seq data from the ENCODE project^28^ (Extended Data Fig. 5a). The strongest association was found for a locus in chromosome 2 with a distal interaction with the *MYCN* promoter region. *MYC*, a paralog *MYCN*, has been convincingly shown to play a crucial role in determining lifespan and shaping various aspects of health and well-being in mammals^29^. We validated this long-range interaction in several datasets, including in situ HiC data from primary cancer cell lines of *MYCN*-driven neuroblastoma, in which we can see a pronounced interaction (Fig. 5c). Furthermore, the found promoter-interacting region overlaps with H3K27ac and H3K4me1 histone marks, suggestive of enhancer activity. A SMC1 Chromatin Interaction Analysis with Paired-End Tag (ChIA-PET) experiment showed that SMC1 sites located at the enhancer/snp and *MYCN* promoter are interacting^30^ (Extended Data Fig. 3e). Once again, this strengthens the case for a physical promoter-enhancer intersection. This link was reinforced by the association of this locus with various cleft lip/palate traits (Extended Data Fig. 3e), for which MYCN deficiency is a known risk factor^31,32^.

## Discussion

The emergence of epigenetic age predictors was a watershed moment in geroscience, propelling the measurement and concept of biological aging into a quantitative era.

Our study attempts to integrate biological mechanisms and quantitative principles to measure epigenetic age. First, we identify and address technical limitations in current epigenetic predictors trained on chronological age. Second, we provide a biologically tangible interpretation of our outputs. This has been a long-held criticism inherent to previous clocks that has hindered the progress in accurately defining the concept of epigenetic speed of aging and in studying the functional mechanisms underlying this process. This study provides a biological interpretation of the speed of aging, at a single-CpG level, defined as the total rate of chemical reactions occurring in cells that result in changes to their methylation states. Two notions central to methylation dynamics naturally emerge in this model: age acceleration, defined as the proportional increase in the speed of aging across sites, and bias, defined as global change in methylation. Our study showed that both acceleration and bias are associated with distinct disease phenotypes. Additionally, we developed a novel batch-correction algorithm which can be used to improve comparison between cohorts or to explore smaller datasets.

We consider our model to be parsimonious in that it uses the minimal set of parameters required to accurately recapitulate observed methylation dynamics; however, a limitation that arises from this approach is the simplification of the mechanism by which cells can gain or lose methylation. Inclusion of enzymatic processes in a more complex model could offer an alternative interpretation of aging on an enzymatic level (also discussed in Supplementary Information Methods 1.3).

Additionally, our model does not account for cell count compositions; however, our analysis of the methylation dynamics observed in CpG sites challenges the widespread hypothesis that methylation changes with age are predominantly influenced by variations in cell-type composition. If this were the case, we should observe clustered patterns of speed of aging across different sites corresponding to different cell types, whereas we observe a continuum. Further, the genomic locus that correlated strongest with age in GS and other studies^6^, ‘cg16867657’ (*ELOVL2*), shows a dynamic range in methylation levels that is inconsistent with cell-type composition changes at old age. Although these remarks are not conclusive, the hypothesis that changes in cell-type composition cannot account for the observed trends in methylation changes at clock CpG sites has already been convincingly argued in previous studies^33^.

The biological relevance of the epigenetic age acceleration and bias measured by our model was substantiated using both phenotype association analysis and GWAS (in combined subsets of GS). Acceleration displayed significant associations with various phenotypes and disease burdens, including diabetes and COPD. These are leading causes of death in high-income countries^34^ and is reflected in our analysis by showing increased risk of mortality associated with increased acceleration. Further we show that bias is a confounder of epigenetic age acceleration that, if unaccounted for, can lead to inaccurate associations, as in the case of alcohol use in first-generation and composite epigenetic clocks. Notably, we see the strongest overlap of our GWAS associations not with the outcomes of first-generation clocks, but with those reported by the composite clock PhenoAge. Although replication in other cohorts should be sought, this highlights that our proposed model captures biologically relevant information.

Downstream functional analysis of the SNPs associated with age acceleration found genetic variants inside a distal enhancer region for the *MYCN* gene. *MYC*, the paralog to *MYCN*, has been associated with aging in a comprehensive study suggesting that the activity of *MYC* plays a crucial role in determining lifespan and affecting various aspects of health and well-being in mammals^29^. *MYCN* as a general cell proliferator in development and the contrasting role in age acceleration make it a candidate gene for antagonistically pleiotropic effects^35^.

## Methods

### Statics and reproducibility

Sample sizes for model inference and validation were determined by the cohort sizes. Randomization and blinding were not applicable during data collection because the study used observational data from population-based cohorts. No participants were excluded in model training and validation. For each continuous phenotype association study, outlier values that were more than 3.5 × s.d. away from the mean were removed.

Randomization and blinding were not applicable during data collection because the study used observational data from population-based cohorts. Statistical tests for model coefficients assumed a normal distribution but this was not formally tested.

### Generation Scotland

#### Overview

GS is a family-based cohort consisting of individuals, aged 18–99 years, living across Scotland. Recruitment took place between 2006 and 2011. The cohort encompasses 5,573 families with a median family size of three (interquartile range of 2–5 members; excluding 1,400 singletons without any relatives in the study). The following analyses were conducted using different subsets of Generation Scotland (*n* = 15,900): GSset1 (*n* = 4,450) the batch-corrected subset GSset2, (*n* = 2,578, unrelated to each other and to GSset1 and processed in separate experimental batches) and the batch-corrected subset GSset3 (*n* = 8,872, processed in separate experimental batches).

Full details on the cohort and baseline data collection have been described previously^36,37^.

#### DNA methylation

Genome-wide DNA methylation was measured from blood samples using the Illumina Infinium HumanMethylationEPIC BeadChip at >850,000 CpG sites. The methylation profiling was carried out in three sets, here referred to as set 1, set 2 and set 3. Set 1 consisted of 4,450 unrelated individuals, who were also unrelated to individuals in set 2. Set 2 consisted of 2,578 individuals who were genetically unrelated to each other at a relatedness threshold <0.025. Set 3 consists of 8,872 individuals with some relatedness to sets 1 and 2 (see GWAS section for GRM approach). Poor performing probes, X/Y chromosome probes and participants with unreliable self-report data or potential XXY genotype were excluded. Full details of DNA methylation quality control steps are detailed under Supplementary Information Methods 6.1.

#### Genotyping

GS samples were genotyped using the Illumina Human OmniExpressExome-8 v.1.0 and 8 v.1.2 BeadChips and processed using the Illumina Genome Studio software v.2011 (Illumina). Quality control steps are outlined in full under Supplementary Information. Duplicate samples, samples with genotype call rate < 0.98 or outlier values on principal-component analysis of genotype data were excluded. SNPs with a call rate < 0.98, minor allele frequency ≤ 0.01 and Hardy–Weinberg equilibrium test with *P* ≤ 1 × 10^−6^ were also removed. Genotype data were imputed using the Haplotype Research Consortium dataset and ~24 million variants were available for analyses^38,39^. There were 15,871 individuals with genotype and methylation data. Full details of genotyping quality control steps are detailed in Supplementary Information Methods 6.2.

#### Phenotyping and health record linkage

GS participants self-reported health and lifestyle data at the study baseline, including lifetime and family history of approximately 20 disease states. Over 98% of GS participants consented to allow access to electronic health records via primary and secondary care records (Readv2 and ICD codes). Data are available prospectively from the time of blood draw, yielding up to 15 years of linkage. Information on mortality and cause of death are updated via linkage to the National Health Service Central Register, which is provided by the National Records of Scotland (data correct as of March 2022).

#### Ethics

All components of GS received ethical approval from the NHS Tayside Committee on Medical Research Ethics (REC reference no. 05/S1401/89). GS has also been granted Research Tissue Bank status by the East of Scotland Research Ethics Service (REC reference no. 20-ES-0021), providing generic ethical approval for a wide range of uses within medical research.

### Associations of other epigenetic clock CpGs with age and smoking

CpG sites were considered naCpGs linear regressions of the form *meth* *~* *age* showed a coefficient of determination *R*^2^ < 0.1. To compute associations between methylation levels and smoking, we created the ‘*weighted smoking*’ variable, which combined information on both how much a participant smoked at any point of in its life, ${norm\_smoke}=(1+\mathrm {{pac}{ks}/{year}})$ and the current smoking status, *curr_smoke*, categorically defined by: (1) currently smoking; (2) stopped within 12 months; (3) stopped more than 12 months ago; or (4) never smoked. More precisely we assumed that the effect of smoking is proportional to *norm_smoke* and fades exponentially with their smoking status, that is ${weighted\_smoke}={norm\_smoke}/\exp ({curr\_smoke})$. In binary definitions of smoking, considering the non-normal distribution of *weighted_smoke*, we used a cutoff of 0.25 in *weighted_smoke* levels, which corresponds to an 85th percentile cutoff from the distribution of *weighted_smoke* values, to define heavy smokers and nonheavy smokers. When calculating smoking associations for the figures, we used this binary definition of smoking and reported the coefficient of smoking from linear regressions for each CpG of the form: ${meth} \sim {weighted\_smoke}+{age}+{sex}$.

### Associations of epigenetic clock CpGs with age and alcohol intake

To assess associations between methylation levels and alcohol use, we transformed the weekly alcohol intake by taking the log of the value plus one. This process allows to recover a normal distribution, given the exponential nature of alcohol use. In binary definitions of alcohol intake, we used a cutoff of an informed mean of 2.3. This number is the mean alcohol intake of participants that reported intake of more than 1 unit per week. We discarded the participants reporting less than 1 unit per week to compute the mean.

### Associations between naCpGs included in Horvath and tissue of origin

To assess the associations between methylation levels and tissue of origin in CpG sites included in Horvath’s clock, we analyzed 3,717 samples from six tissues (cerebellum, breast, liver, kidney, whole blood and saliva) extracted from the EWAS Datahub^18^.

First, we used the combined dataset to compute linear regressions of the form *meth* *~* *age* for all CpGs included in Horvath’s epigenetic clock. We found that 80% (*n* = 288) of all sites included in Horvath (*n* = 354) showed an *r*^2^ < 0.1 and were considered naCpGs.

We then checked for correlations between methylation levels and tissue of origin using a one-way analysis of variance. All naCpG sites showed differential methylation levels across tissues (*P* < 1 × 10^−5^).

### Bootstrapping linear regression models

To show the decline in associations with increasing training size cohorts, we selected random training cohorts with set sizes and test sets (*n* = 890 or 20% of GS_set1_) from GS_set1_. We trained LASSO models with fivefold cross validation to allow optimization of the selected CpG sites and regularization levels.

We then computed acceleration as the residual from the model prediction and chronological age and inferred its association with *weight_smoke* on the test set.

We bootstrapped linear regression models with increasing proportions of smokers (categorically defined as *weight_smoke* > 0.25) similarly. Here training sets had a fixed size (*n* = 700) limited by the number of smokers.

### Variance in methylation levels across age bins

To evaluate the increase in variance of methylation levels with age in GS_set1_, we first selected all sites for which methylation levels correlated with age (*R*^2^ > 0.1). Then we split all participants between 18 and 80 years across five equally spaced age bins (each bin showed an age range of 12.4 years). To promote a fair comparison, and avoid under or over estimation, of variance across bins we randomly selected 294 participants from each bin, the lowest number of participants in any bin, which ensured equal representation of samples in bins.

### Spearman selection of CpG sites

To avoid numerical errors during model fitting in GS_set1_ we discarded CpG sites with NaN methylation values (*n* = 6) and replaced methylation levels of 0 and 1 by 0.0001 and 0.9999, respectively. Next, we fitted linear regression.

We fitted linear regression models of the form *meth* *~* *age* on the remaining sites (*n* = 773,854). To maximize the Spearman correlation, *ρ*, between methylation changes and chronological age in sites used for model training, and to avoid the presence of naCpG sites in our model, we filtered all CpG sites showing a low coefficient of determination, *ρ*^2^ < 0.2. The remaining sites (*n* = 1,870) were taken forward for model comparison.

### Biological modeling of methylation dynamics at a single CpG site level

We now present an overview of the key results necessary to understand the derivation and interpretation of the proposed mathematical model of methylation dynamics. An exhaustive description and precise mathematical derivations can be found in the Supplementary Methods.

To model the evolution of methylation dynamics with time, in a single CpG site, we considered a minimal model of transitions between two states. The proposed model directly relates to the early work of Markov in 1905 and was used for the first time to model the dynamics of methylation^16^ and more recently in the context of aging^17^. In this model, cells can be in either an unmethylated (U) or methylated (M) state and can transition from one state to the other at rates *V*_U_ and *V*_M_ (Fig. 2b).

The mean evolution of this system as a function of time, $t$, is given by

$$\mu(t)=\eta +{e}^{-\omega t}(p-\eta ),$$

where $\omega ={\nu }_{\mathrm {U}}+{\nu }_{\mathrm {M}}$ corresponds to the total rate of transitions, $p$ the proportion of methylation cells at initial time *t* = 0 and $\eta ={\nu }_{U}/\omega$ to the proportion transitions associated with a gain in methylation (Fig. 1a). Notice that the definition of speed of epigenetic aging at a single CpG site, $\omega$, emerges naturally, while the directionality of these changes is independent from the speed, given by the initial state of the system $p$ and the final state $\eta$.

The evolution of the variance of this system can be similarly described as a function of time,

$$\begin{array}{l}{\sigma }^{2}(t)=\frac{{\eta }_{U}{\eta }_{M}}{S}+{e}^{-\omega t}\frac{{{\eta }_{U}}^{2}(1-p)+p{{\eta }_{M}}^{2}-{\eta }_{U}{\eta }_{M}}{S}\\\qquad\qquad+\,{e}^{-2\omega t}\left(\frac{c}{{S}^{2}}-\frac{{{\eta }_{U}}^{2}(1-p)+p{{\eta }_{M}}^{2}}{S}\right),\end{array}$$

where *S* corresponds to the system size and *c* is the initial variance at the cell level.

Although the distribution for the evolution in time of this system is not analytically solvable, it is well approximated by a Beta distribution conditional on the mean and variance derived above. We can therefore model the probability of seeing a methylation level *m*_i_ in site i in an individual aged *t* by

$$P({m}_{i}{\rm{|}}t)={Bet}{a}_{p.d.f.}({m}_{i},\mu_i(t),\,{\sigma_i }^{2}(t)),$$

where Beta p.d.f. denotes the probability density function of a Beta distribution and the mean and variance are adapted to each site. We use this probability definition to find the optimal parameters for each site using all cohort methylation observations. That is, the parameters that maximize

$$P({M}_{i})=\prod _{({{m}_{i}}^{j},{t}^{j})\in {M}_{i}}{Bet}{a}_{p.d.f.}({{m}^{j}}_{i},\mu_i({t}^{j}),\,{\sigma_i }^{2}({t}^{j})),$$

where ${M}_{i}=({{m}_{i}}^{j},{t}^{j})$ corresponds to the pair of methylation value and age of each individual in the cohort. We approximated the full posterior distribution, using a Markov chain Monte Carlo algorithm and extracted the maximum a posteriori (MAP) values of the parameters for each site using the PyMC Python package.

To ease interpretation of our results, we take the log_2_ transformation of the original acceleration parameter, centering the cohort average at 0, and report the MAP estimates for both acceleration and bias.

### Model comparison

Similarly, we fitted a probabilistic linear model of the form

$$P({M}_{i})=\prod _{({{m}_{i}}^{j},{t}^{j})\in {M}_{i}}{Nor}{m}_{p.d.f.}({m}_{i},{a_i t}+b_i,{c_i t}+d_i),$$

and inferred the posterior distribution and performed a model comparison using the PyMC Python package^40^. Model comparison approximated the expected log-predictive density (ELPD) of both models on each site using an approximation of leave-one-out cross validation (LOO-CV) based on the Pareto-smoothed importance sampling. The higher ELPD value on each site highlights the favored model to explain the observed dynamics. As the evolution of methylation across CpG sites is assumed to be independent of each other, we compute the overall ELPD for either model by summing the reported value on all fitted sites.

### Saturation filtering

Further quality control measures were taken to ensure that only sites that capture the full expected dynamics of methylation changes with time are retained for further modeling (Extended Data Fig. 2a).

We observed that the methylation dynamics are constrained between 0 and 1. This reduces the possibility to observe deviations from the mean in sites approaching these boundaries. We therefore dropped sites for which the 95% CI predicted by our model reached a threshold of either 0.005 and 0.95, at either birth or 90 years. A total of 204 sites were observed to display this phenomenon.

Further, we noticed that the above-mentioned phenomenon might occur at different thresholds, due to different batch-correction processes; however, in these sites, we should see a plateau in the evolution of the methylation dynamics. We therefore computed the derivative of the mean methylation dynamics for each site, $m{\prime} (t)=\omega {e}^{-\omega t}(\eta -p)$, and valued it at *t* = 90. This value is a measure of our distance to steady state at old age, ${ds}=m{\prime} (90)$, at old age. We then filtered all sites with low distances to steady state, *ds* < 0.001 (*n* = 654).

Overall, a total of 1,160 sites were taken forward for acceleration and bias modeling.

### Acceleration and bias model

First, we define the probability of observing a methylation pattern in an individual by comparing its methylation levels to those obtained from cohort fitting on each CpG site. That is, if an individual of age *t* has methylation levels $\{{m}_{i}{\}}_{i\in I}$ on a set of CpG sites $I$, we define its probability of observation as

$$P(\{{m}_{i}\}_{i\in I},t)={\prod }_{i\in I}\quad{Bet}{a}_{p.d.f.}({m}_{i},\mu_i(t),\,{\sigma_i }^{2}(t)),$$

where $m(t)$ and ${\sigma }^{2}(t)$ are defined as above, using the MAP values for all site parameters in the model obtained from the cohort fitting. We then consider two extra parameters *α* and *β* in our model. Mathematically, we multiply the speed of reactions *V*_U_ and *V*_M_ uniformly across all sites by a factor *α*. Notice that this translates in a proportional increase of the total speed of reaction, which becomes $\alpha {\omega }_{i}$ for each CpG site *i*. Parameter α therefore represents a uniform increase in the average speed of epigenetic aging across all measured sites. Analogously, parameter *β* modifies uniformly all sites shifting the mean intercept, but not disturbing the expected speed of change. These two parameters modify the mean evolution of methylation on for each site as follows:

$$\mu_i(t,\alpha ,\beta )=\eta_i +{e}^{-\alpha \omega_i t}(p_i-\eta_i )+\beta .$$

Details on how these parameters modify the evolution of the variance can be found in the Supplementary Methods Section 4. We can then compute the probability of observing an individual conditional on a given acceleration and bias

$$P(\{{m}_{i}\}_{i\in I},t{\rm{|}}\alpha ,\beta )={\prod }_{i\in I}\quad{Bet}{a}_{p.d.f.}({m}_{i},\mu_i(t,\alpha ,\beta ),\,{\sigma_i }^{2}(t,\alpha ,\beta )).$$

We then infer the posterior distribution of parameters *α* and *β* and compute the MAP values that maximize the probability of observing the methylation levels in an individual across sites using the PyMC Python package.

### Downsampling

To determine the optimal amount of CpG sites included for the inference of the acceleration and bias of each individual we conducted a downsampling experiment. We inferred acceleration and bias using increasing numbers of CpG sites, ordered decreasingly according to their absolute correlation with age. We then computed the absolute difference between the inferred values for each fit to infer the stability of our predictions as a function of the sites included in the model. We found that there are no benefits in including more than 250 sites (Extended Data Fig. 1b).

### Order of magnitude difference analysis

To quantify the differences in the predicted age accelerations between a model with masked data and a model trained with all data, we computed the differences between the order of magnitude of the absolute change between model predictions and the order of magnitude of accelerations predicted by the unmasked model,

$${OMD}=\log \left|{ac}{c}_{{mask}}-{ac}{c}_{{full}}\right|-\log \left|{ac}{c}_{{full}}\right|.$$

Negative values of the order of magnitude suggest that the differences between the mask model and the full model are negligible.

### Global hypo–hyper methylation test

We tested the robustness of our accelerated person model by transforming the training GS_set1_ cohort, by applying a global hypo or hyper methylation to all the data points. Then we inferred the acceleration and bias values on this transformed dataset using the site parameters inferred from the not transformed dataset (Extended Data Fig. 1d). As a benchmark, we also predicted the age acceleration using the Horvath epigenetic clock which shows little robustness against these type of methylation transformations.

### Site batch correction

When applying our model to external datasets, we do not retrain the biological model on the new data. Instead, we applied an offset to the evolution of the mean $m(t)$, for each site, inferred using our reference dataset GS_set1_. Mathematically, for each site we find the offset, ${o}_{i}$, which maximizes the probability of observing

$$P({M}_{i})={\prod }_{({{m}_{i}}^{j},{t}^{j})\in {M}_{i}}\quad{Bet}{a}_{p.d.f.}(m,\mu_i(t)+{o}_{i},\,{\sigma_i }^{2}(t)).$$

The full mathematical description of our algorithm can be found in Supplementary Methods section 5. We benchmarked our results on the Hannum dataset by comparing a fully retrained model on this dataset and the model trained on GS_set1_ applied both with and without batch correction. We found that offset does not have a clear directionality across sites and should therefore be applied separately for each CpG site considered in the model.

### Down syndrome analysis

We used the control group in the Down syndrome dataset^24^ to fit our batch-correction algorithm and inferred acceleration and bias parameters for the control (*n* = 58) and disease (n = 29) groups. We then fitted a logistic regression model of the form ${disease} \sim {scale}({acceleration})$.

### Association analysis

We utilized the linked health data records of GS_set1_, GS_set2_ and GS_set3_ (*n* = 15,900) to perform association studies grouped into three categories associated with the nature of the studied traits: continuous, disease and mortality.

For each continuous phenotype, outlier values that were more than 3.5 × s.d. away from the mean were removed before analysis. We also applied a log transformation to the body mass index to normalize the data. Finally, we used log transformations on alcohol consumption to reduce skewness in their distributions. This was carried out by applying a log(units + 1) transformation. We quantified the continuous trait of smoking using our developed *weighted_smoke* parameter (described above).

#### Statistical analysis

Linear regression models of the form ${scale}({phenotype}) \sim$ ${scale}(\alpha )+{scale}(\beta )+{age}+{sex}$ were used to examine the association between continuous traits and the acceleration and bias measurement. Linear regression models of the form ${scale}({phenotype}) \sim$ ${scale}({clock})+{age}+{sex}$ were used to examine the association between continuous traits and the four epigenetic clocks. Scaling is performed to standardize the data to a mean of zero and a variance of one. Logistic regression was used to test the association between categorical disease phenotypes and acceleration and bias. We fitted a Cox proportional hazards regression to examine whether our measures of epigenetic biological aging were associated with incidences of all-cause mortality. All of the results were corrected using the FDR method.

### GWAS and functional analysis

Genotype–phenotype association analyses were performed using a linear mixed model GWAS implemented in fastGWA GCTA^41^. A sparse GRM approach is used to adjust for sample relatedness. Overall, 15,871 overlapping individuals with nonmissing genotype and phenotype data were included. Variants with MAF < 0.01 or missingness rate > 0.1 were excluded from the analysis. Preliminary functional annotation was performed using FUMA^42^ and COJO^41^. Genomic risk loci were defined around significant variants (*P* < 5 × 10^−8^) and included all variants correlated (*R*^2^ > 0.1) with the lead variant. We used LDtrait^43^ to search for phenotypes associated with SNPs in linkage disequilibrium with the four lead SNPs (*R*^2^ > 0.1) and within 1 Mb. LDtrait reports association data from the GWAS catalog^25^. All coordinates in this study were based on human reference genome assembly GRCh37/hg19 (see website in Framework Implementation). Gene annotations were based on GENCODE annotation release 39 (see website listed in ‘Framework implementation’).

### Framework implementation

The inference of the posterior distribution of model parameters was implemented in Python version 3.9 with dependencies on PyMC 5.0.2 41, Numpy 1.24.1 45, Anndata 0.8.0 46 and Pandas version 1.5.3 47. Cox proportional hazards regression was done using the survival package version 3.4.0 48, while linear and logistic regression were done using the stats base package under R base version 4.2.2. GWAS summary statistics was generated using GCTA version 1.93.2. Functional analysis of the GWAS results was done using FUMA version 1.5.1. Human reference genome assembly used for GWAS: http://www.ncbi.nlm.nih.gov/assembly/2758/. Gencode annotation researle 39: https://www.gencodegenes.org/human/release_39.html.

### Reporting summary

Further information on research design is available in the Nature Portfolio Reporting Summary linked to this article.

## Supplementary information

Supplementary Information

Reporting Summary

Supplementary Tables Supplementary Table 1. Association analysis results for continuous phenotypes estimated using linear regression models. Supplementary Table 2. Association analysis results for self-reported disease phenotype estimated using logistic regression models. Supplementary Table 3. Association analysis results for all-cause mortality estimated using Cox proportional hazards models. Supplementary Table 4. Summary statistics of the genomic risk loci showing genome-wide significant level of association with acceleration. *P* values shown here are unadjusted and two-tailed. Supplementary Table 5. Lead SNPs associated with acceleration, analyzed on the FUMA platform. *P* values shown here are unadjusted and two-tailed. Supplementary Table 6. GWAS catalog look up results after distance-based (±500 kb) and LD (*r*^2^ > 0.1) pruning. Supplementary Table 7. acceleration and bias measure from training data (GS set1).

## Data Availability

According to the terms of consent for GS participants, access to data must be reviewed by the GS Access Committee. Applications should be made to access@generationscotland.org. Applications are reviewed usually within 6–8 weeks of submission, but typically sooner. Applicants are notified of the decision no later than 2 weeks after the meeting. The dataset ‘Sample information of DNA methylation profiles of male and female in 24 tissues’ used to check the associations for tissue-specific sites is publicly available at the EWAS Datahub at https://download.cncb.ac.cn/ewas/datahub/download/sex_methylation_v1.zip. The Down syndrome dataset is publicly available in the Gene Expression Omnibus under accession no.GSE52588. The Hannum dataset is publicly available in the Gene Expression Omnibus under accession no. GSE40279.

## Code availability

All code used in this manuscript is available at https://github.com/zuberek/probage. The inference of the posterior distribution of model parameters was implemented in Python v.3.9 with dependencies on PyMC v.5.0.2 (ref. ^40^), Numpy v.1.24.1 (ref. ^44^), Anndata v.0.8.0 (ref. ^45^) and Pandas v.1.5.3 (ref. ^46^). Cox proportional hazards regression was conducted using the survival package v.3.4.0 (refs. ^47,48^), while linear and logistic regression were carried out using the stats base package under R base v.4.2.2. GWAS summary statistics were generated using GCTA v.1.93.2. Functional analysis of the GWAS results was conducted using FUMA v.1.5.1. The human reference genome assembly used for GWAS is at http://www.ncbi.nlm.nih.gov/assembly/2758/. GENCODE annotation release 39 is at https://www.gencodegenes.org/human/release_39.html.
